# Cyberbullying in adolescents: the next transition frontier

**DOI:** 10.1186/1546-0096-12-S1-P106

**Published:** 2014-09-17

**Authors:** Derek Deely, Deirdre Carroll, Emma MacDermott, Orla Killeen

**Affiliations:** 1 National Centre for Paediatric Rheumatology, Our Lady's Children's Hospital, Dublin 12, Ireland

## Introduction

Cyberbullying, particularly among adolescents, is an emerging issue within our society. Cyberbullying shares similar characteristics with traditional bullying such as repetition, power imbalance and intention. However, it differs in the fact that it is anonymous and rapid, with its victims unable to escape. The effects of cyberbullying on young people may have serious negative affects on their confidence, self-esteem, and emotional and mental wellbeing.

## Objectives

To assess the incidence, experience, knowledge and beliefs on cyberbullying.

## Methods

A convenience sample of 14 patients completed an on-line questionnaire. All participants attend the National Centre for Paediatric Rheumatology, Dublin.

## Results

14 participants: 6 Male, 8 Female. Median age = 14 years (13-18). 100% have daily access to the internet (average daily use, 3.8 hours).100% have a profile page on 1 or more social networking sites (SNS). 45% do not have their SNS protected i.e. maximum privacy settings. 100% were familiar with the term “cyberbullying”. 83 % believed that social networking sites were the most common forum used to bully.

Participants were asked if they had heard of the following categories of cyberbullying (Table 1)

**Table 1:** 

Flaming (online fights)	83%
**Harassment (Repeated, malicious messages)**	100%

**Denigration (Posting rumours)**	58%

**Masquerading (The bully pretends to be the target)**	42%

**Outing (Sharing someone’s secrets)**	58%

**Trickery (Talking someone into revealing secrets, then sharing it online)**	58%

**Exclusion (Intentionally excluding someone from an online group)**	42%

**Cyber threats**	83%

**Cyber stalking**	58%

67% of participants believed harassment was the most common type of cyberbullying followed by flaming (17%), cyber threats (8%) and cyber stalking (8%).

When asked if they had ever been a victim of cyberbullying, 33% admitted that they had. Of these 100% stated they had been harassed while 1 reported they were victims of flaming with 1 subjected to outing. All those who admitted to have been bullied in the past stated they did nothing about it.

2 respondents admitted to bullying other people online previously. When asked why, both stated it was in response to being threatened / harassed first. 58% stated they had been contacted by a stranger via the internet. Of these 29% reported that the stranger did try to arrange to meet them in person. 1 participant provided the stranger with false information about themselves and 1 gave their mobile phone number.

56% believed that health care professionals play an important role in preventing cyberbullying. 100% reported that if they were being bullied online that they would report it to a doctor or a nurse.

## Conclusion

Awareness of health care professionals on the challenges associated with internet use is required in order to promote the safety and health of adolescents in cyberspace.

## Disclosure of interest

None declared.

